# Clinical Utility of Gait Speed Indices for Identifying Sarcopenia in Older Adults with Type 2 Diabetes

**DOI:** 10.3390/geriatrics11020046

**Published:** 2026-04-14

**Authors:** Kensaku Kashima, Rie Nishimura, Hisashi Sugano, Shimpei Fujimoto

**Affiliations:** 1 Department of Rehabilitation, Kochi Health Sciences Center, Kochi 781-8555, Japan; kensaku_kashima@khsc.or.jp (K.K.); rie_nishimura@khsc.or.jp (R.N.); 2 Department of Metabolism and Endocrinology, Kochi Health Sciences Center, Kochi 781-8555, Japan; dr-hisashi@siren.ocn.ne.jp; 3 Department of Endocrinology, Metabolism, and Nephrology, Kochi Medical School, Kochi University, Kochi 783-8505, Japan

**Keywords:** type 2 diabetes, sarcopenia, gait speed, gait speed reserve, diagnostic performance

## Abstract

**Background/Objectives**: This study aimed to compare the diagnostic performance of usual gait speed (UGS), maximal gait speed (MGS), and gait speed reserve (GSR) for identifying sarcopenia in older adults with type 2 diabetes (T2D), and to examine whether combining gait indices improves diagnostic performance. **Methods**: This cross-sectional study included 117 older adults with T2D hospitalized for glycemic control. UGS and MGS were measured in the central 10-m section of a 16-m course, which included 3-m acceleration and deceleration zones on either side. GSR was calculated as the difference between MGS and UGS. Sarcopenia was diagnosed according to the AWGS 2025 criteria. Multivariable logistic regression was used to examine the associations between each gait index and sarcopenia. Diagnostic performance was evaluated using receiver operating characteristic (ROC) curve analysis, and the usefulness of a combined criterion based on UGS and GSR was also assessed. **Results**: Sarcopenia was identified in 32 participants (27.4%). UGS, MGS, and GSR were each associated with sarcopenia independently of clinical factors. UGS showed the highest sensitivity (90.6%), suggesting its potential usefulness as a screening measure, whereas GSR showed the highest specificity (83.5%), suggesting its potential usefulness in reducing false positives when identifying sarcopenia. In the ROC analysis of the three gait-related indices, the area under the curve (AUC) for MGS was the highest (0.79), but it was not significantly different from the AUC for UGS. The combined criterion of UGS < 1.07 m/s and GSR < 0.21 m/s improved specificity and yielded a high positive likelihood ratio (PLR = 6.5). **Conclusions**: UGS, MGS, and GSR each exhibit distinct diagnostic characteristics for sarcopenia. UGS may be useful for screening, whereas GSR may help improve specificity. In particular, combining UGS and GSR may provide complementary diagnostic information for identifying sarcopenia in older adults with T2D.

## 1. Introduction

With the global rise in diabetes and the rapid aging of populations, sarcopenia has become a major concern in the clinical management of individuals with type 2 diabetes (T2D) [1]. A narrative review by Izzo et al. reported that sarcopenia is approximately 1.5–1.6 times more prevalent in individuals with T2D than in those without diabetes, highlighting its clinical importance as a common diabetes-related comorbidity [2]. Sarcopenia is characterized by loss of skeletal muscle mass along with declines in muscle strength and physical performance, which may have a negative impact on both physical and psychosocial health in this population [2].

T2D is associated with significantly lower muscle strength and quality [3] and with accelerated decline in lower-limb muscle strength and quality [4] compared with individuals without diabetes. Volpato et al. also demonstrated that people with T2D have poorer lower-limb muscle function and slower gait speed than people without T2D [5]. Lower muscle strength and quality may contribute to disability in activities of daily living (ADL) and instrumental activities of daily living (IADL). Meta-analyses have shown that T2D increases the risk of falls, ADL disability, and IADL disability by approximately 1.6-, 1.8-, and 1.7-fold, respectively [6,7].

The diagnostic criteria for sarcopenia have been widely defined by the European Working Group on Sarcopenia in Older People (EWGSOP) [8] and the Asian Working Group for Sarcopenia (AWGS) [9]. The updated AWGS 2025 criteria require the assessment of skeletal muscle mass and muscle strength for the diagnosis of sarcopenia [9]. Although gait performance is included as a component of physical function, its independent diagnostic value for sarcopenia remains unclear.

Meanwhile, the assessment of muscle mass and strength requires specialized equipment, which limits its feasibility in routine clinical and community settings. Although simple questionnaires such as the SARC-F are available, their diagnostic accuracy remains suboptimal [10,11]. Therefore, establishing simple, reproducible, and clinically applicable methods to diagnose sarcopenia is important.

Gait speed is a widely accepted indicator for both frailty and physical functional decline, valued for its prognostic utility and ease of measurement [12]. Usual gait speed (UGS) has been included in previous AWGS diagnostic criteria and definitions of frailty [13,14], and has been used as a screening indicator. In contrast, maximal gait speed (MGS) correlates more strongly than UGS with knee extension strength [15], declines more markedly with age, and is associated with mortality risk, particularly cardiovascular mortality [16,17]. It may also influence the classification of sarcopenia severity and is more closely related to functional outcomes such as the Barthel Index, SARC-F scores, and fall history [18].

Furthermore, gait speed reserve (GSR), defined as the difference between MGS and UGS, has been proposed as an index that integrates information from both measures [19,20,21,22,23,24]. GSR reflects the capacity to increase gait speed beyond habitual levels and is considered an indicator of physiological reserve. Previous studies have shown that GSR is associated with frailty-related outcomes [19], cognitive function [20], lower-limb motor coordination [21], and cardiovascular risk [22]. In community-dwelling older adults, reserve-based gait indices derived from usual and maximal gait speed showed a moderately useful positive likelihood ratio (PLR) of 6.6 for identifying frailty [23], and in patients undergoing hemodialysis, higher GSR was independently associated with lower all-cause mortality (HR = 0.44) [24]. However, evidence regarding the diagnostic performance of GSR for identifying sarcopenia, particularly in individuals with T2D, remains limited.

Despite these findings, the roles of MGS and GSR in the diagnosis of sarcopenia have not been well established in T2D. Therefore, this study aimed to investigate the clinical utility of MGS and GSR for identifying sarcopenia in older adults with T2D.

## 2. Materials and Methods

### 2.1. Participants and Ethical Considerations

This cross-sectional study included 176 individuals with T2D who were admitted to Kochi Health Sciences Center between June 2016 and December 2024 for diabetes management. The diagnosis of T2D was based on the WHO criteria [25]. We excluded those aged <65 years (*n* = 51), those with dementia (*n* = 2), those with motor paralysis due to central nervous system disorders (*n* = 2), and those requiring assistance for walking (*n* = 4). Consequently, 117 individuals were included in the final analysis.

Clinical variables, including diabetes duration, HbA1c, renal function (estimated glomerular filtration rate [eGFR]), diabetes-related complications (neuropathy, retinopathy, and nephropathy), and comorbidities (hypertension, dyslipidemia, cardiovascular disease, and cerebrovascular disease), were extracted from electronic medical records. HbA1c and eGFR were obtained from blood tests performed at admission or within one week prior to admission. These variables were collected to characterize the clinical background of the participants.

This study was conducted in accordance with the Declaration of Helsinki and approved by the Ethics Committee of Kochi Health Sciences Center (protocol code 241056; approval date: 20 November 2024). This study complied with the Act on the Protection of Personal Information and the Ethical Guidelines for Medical and Health Research Involving Human Subjects in Japan. Informed consent was obtained using an opt-out procedure.

### 2.2. Assessment of Muscle Strength and Physical Function

Handgrip strength (HGS) was measured using a digital dynamometer (GRIP-D TKK5401; Takei, Niigata, Japan). Participants were instructed to maintain a standing position with the elbow extended or slightly flexed. Two trials were performed for each hand, and the highest value obtained was used for analysis.

UGS and MGS were measured on a 16-m walkway that included 3-m acceleration and deceleration zones at each end. Participants walked wearing their usual footwear without assistive devices. The time required to traverse the central 10-m section was measured using a handheld stopwatch by a trained examiner. For UGS, participants were instructed to walk “at their usual pace,” whereas for MGS, they were instructed to walk “as fast as possible without running.” Each condition was assessed twice, and the faster value was used for analysis. Previous studies have shown good reliability for usual and fast gait speed tests in community-dwelling older adults (ICC 0.72–0.98) [26]. GSR was calculated as MGS minus UGS.

Skeletal muscle mass was measured using a multifrequency bioelectrical impedance analyzer (InBody 770; InBody Co., Ltd., Seoul, Republic of Korea). Measurements were obtained at rest in a standing position, in the absence of clinically apparent edema, according to standardized clinical procedures. All measurements were performed under consistent conditions using the same device. Previous studies have shown excellent test–retest reliability for multifrequency bioelectrical impedance analysis (BIA) using the InBody 770 (ICC 0.987–0.995) [27]. In addition, in older adults with T2D, skeletal muscle mass index (SMI) measured by InBody 770-based multifrequency BIA showed high agreement with that measured by dual-energy X-ray absorptiometry (ICC = 0.965) [28]. Appendicular skeletal muscle mass (kg) was divided by height squared (m^2^) to calculate the SMI (kg/m^2^).

### 2.3. Diagnosis of Sarcopenia

Sarcopenia was evaluated with reference to the recommendations of the AWGS 2025 [9]. In this study, sarcopenia was defined as the coexistence of low muscle mass and low muscle strength. Low muscle mass was determined using the SMI, with cutoff values of <7.0 kg/m^2^ for men and <5.7 kg/m^2^ for women. Low muscle strength was defined as HGS <28 kg for men and <18 kg for women.

### 2.4. Statistical Analysis

All statistical analyses were performed using EZR version 4.5.1 (Jichi Medical University Saitama Medical Center, Saitama, Japan), a graphical user interface for R (The R Foundation for Statistical Computing, Vienna, Austria) [29]. A two-sided *p*-value < 0.05 was considered statistically significant.

Continuous variables are presented as median (interquartile range), and categorical variables as frequency (%). Between-group comparisons were performed using the Mann–Whitney U test for continuous variables and the chi-square test or Fisher’s exact test for categorical variables, as appropriate.

In the multivariable logistic regression analyses, the presence of sarcopenia (yes/no) was used as the dependent variable. Each gait index (UGS, MGS, or GSR) was entered as the primary independent variable in separate models. The models were adjusted for age, sex, BMI, and diabetic neuropathy. Age, sex, and BMI were selected based on clinical relevance and prior literature [30], and diabetic neuropathy was additionally included because it was the only diabetes-related variable that differed significantly between groups.

HGS and SMI were excluded because they are components of the diagnostic definition of sarcopenia. To avoid multicollinearity, UGS, MGS, and GSR were evaluated in separate models. Gait speed variables were modeled as continuous variables, and odds ratios were expressed per 0.1 m/s increase to facilitate clinical interpretation.

Diagnostic performance of each gait index for identifying sarcopenia was evaluated using receiver operating characteristic (ROC) curve analysis. Sensitivity, specificity, and the area under the curve (AUC) were calculated, and differences between AUCs were assessed using the DeLong test. Optimal cutoff values were determined using the Youden index, and positive predictive value (PPV), negative predictive value (NPV), diagnostic accuracy, PLR, and negative likelihood ratio (NLR) were subsequently calculated. Diagnostic performance for the combined criterion based on UGS and GSR was evaluated using cross-tabulation analysis with ROC-derived cutoff values.

## 3. Results

### 3.1. Participant Characteristics

Compared with the non-sarcopenia group, the sarcopenia group was significantly older (*p* < 0.001). The prevalence of diabetic neuropathy was also higher in the sarcopenia group (*p* = 0.036). In addition, BMI, SMI, HGS, UGS, MGS, and GSR were significantly lower in the sarcopenia group (all *p* < 0.001). No significant differences were observed in sex, diabetes duration, HbA1c, or other comorbidities (Table 1).

### 3.2. Predictive Factors for Sarcopenia Diagnosis

Because diabetic neuropathy was the only diabetes-related variable that differed significantly between groups, it was included as a covariate in the multivariable logistic regression analyses. After adjustment for age, sex, BMI, and diabetic neuropathy, UGS, MGS, and GSR were each independently associated with sarcopenia (Table 2).

### 3.3. ROC Analyses and Diagnostic Performance

**Figure 1:**
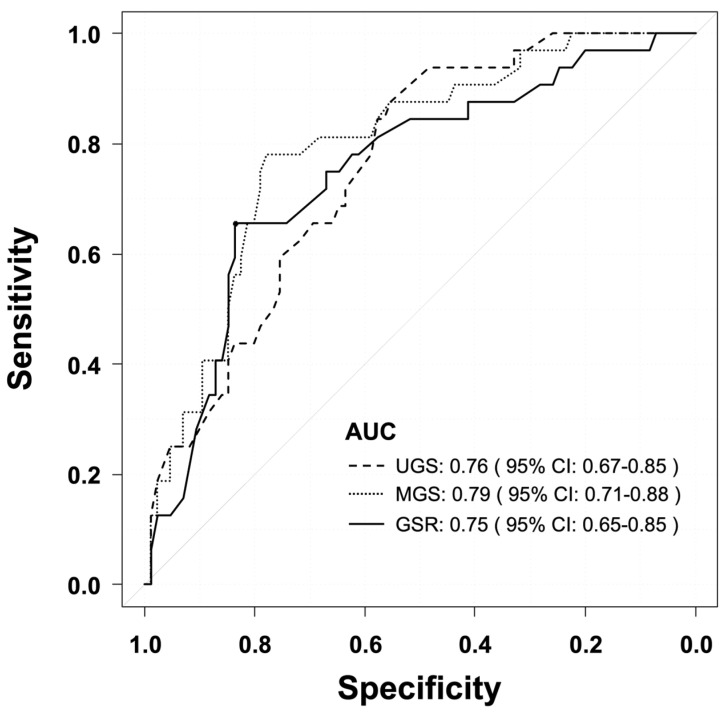
ROC curves for UGS, MGS, and GSR in identifying sarcopenia. The AUC values were 0.76 (95% CI: 0.67–0.85) for UGS, 0.79 (95% CI: 0.71–0.88) for MGS, and 0.75 (95% CI: 0.65–0.85) for GSR. There was no significant difference between UGS and MGS (DeLong test, *p* = 0.190). UGS, usual gait speed; MGS, maximal gait speed; GSR, gait speed reserve; AUC, area under the curve.

ROC analysis showed AUC values of 0.76 (95% CI: 0.67–0.85) for UGS, 0.79 (95% CI: 0.71–0.88) for MGS, and 0.75 (95% CI: 0.65–0.85) for GSR (Figure 1). In the ROC analysis of the three gait-related indices, the AUC for MGS was the highest, but it was not significantly different from the AUC for UGS (DeLong test, *p* = 0.190).

The optimal cutoff values determined by the Youden index were 1.07 m/s for UGS, 1.28 m/s for MGS, and 0.21 m/s for GSR.

UGS showed the highest sensitivity and NPV, suggesting its usefulness as a screening indicator for sarcopenia. MGS demonstrated a good balance between sensitivity and specificity. GSR showed the highest specificity among the individual indices.

In addition, the combined criterion of UGS < 1.07 m/s and GSR < 0.21 m/s further improved specificity, PPV, and PLR and showed high diagnostic accuracy (Table 3).

## 4. Discussion

The AWGS 2025 criteria for diagnosing sarcopenia include HGS [9]. However, HGS has only a weak correlation with lower-limb muscle strength [31], and its ability to represent overall muscle strength remains controversial. The Global Leadership Initiative in Sarcopenia (GLIS), which recently proposed a conceptual definition of sarcopenia, recommends including muscle-specific strength in diagnostic criteria [32]. However, because large-scale data on muscle-specific strength, including quadriceps strength, remain limited, HGS—an index for which substantial data are available—is currently used as a key diagnostic component. Despite this limitation, sarcopenia was diagnosed in the present study according to the AWGS 2025 criteria, which are considered appropriate for Asian populations.

First, to identify potential confounding factors among the older participants with T2D in this study, we compared the putative risk factors between the sarcopenia group and the non-sarcopenia group. Age, BMI, and diabetic neuropathy differed significantly between the two groups, and these findings were broadly consistent with a recent systematic review and meta-analysis of Asian adults with T2D [30]. In particular, the higher prevalence of diabetic neuropathy in the sarcopenia group was also consistent with that report [30].

Because these factors could act as potential confounders, we performed logistic regression analyses with the presence or absence of sarcopenia as the dependent variable and these factors together with the gait-related indices as independent variables. As a result, all three gait-related indices remained significantly associated with sarcopenia after adjustment for these potential confounders, and the upper bounds of the 95% confidence intervals for the odds ratios were all 0.75 or lower. These findings indicate that all three gait-related indices were independently associated with sarcopenia after adjustment for potential confounders. Their relative diagnostic utility was then evaluated using ROC analysis.

In the ROC analysis of the three gait-related indices, the AUC for MGS was the highest (0.79), but it was not significantly different from the AUC for UGS. UGS showed the highest sensitivity (90.6%), suggesting its potential utility as a screening marker, whereas GSR showed the highest specificity (83.5%), indicating potential value when greater diagnostic certainty is required. In addition, the combined criterion of UGS < 1.07 m/s and GSR < 0.21 m/s improved specificity to 89.4% and yielded a positive likelihood ratio of 6.5, suggesting potential utility for more specific identification of sarcopenia in older adults with T2D. Taken together, these findings suggest that gait-related indices may provide clinically useful and complementary information for identifying sarcopenia in this population.

These findings are also consistent with previous studies suggesting that MGS may reflect lower-limb muscle function more strongly than UGS. Bohannon et al. reported that lower-limb muscle strength correlates more strongly with MGS than with UGS [15]. Similarly, Haigis et al. demonstrated that MGS was significantly associated with ADL-related scales, screening indicators for sarcopenia, and fall history, whereas UGS showed no such associations [18]. Taken together, these findings suggest that MGS may reflect lower-limb muscle strength and functional reserve more strongly than UGS.

GSR is defined as the difference between maximal and usual gait speeds and represents the ability to increase walking performance beyond habitual levels. Therefore, GSR can be interpreted as an indicator of physiological or functional reserve capacity. Previous studies have reported that reduced gait reserve is associated with frailty and disability in ADL [19]. Our findings suggest that reduced gait reserve reflects diminished physical reserve in individuals with sarcopenia.

Although the AUC of GSR was slightly lower than those of UGS and MGS, it demonstrated the highest specificity for identifying sarcopenia among older adults with T2D in the present study. This finding suggests that GSR may be more useful for improving specificity than for screening purposes. The optimal cutoff value of GSR identified in this study was 0.21 m/s, which likely represents reduced gait reserve capacity in individuals with sarcopenia. We also examined the potential value of combining UGS and GSR. Because UGS is sensitive for screening and GSR is more specific by reflecting reduced physiological reserve, their combination may provide a practical strategy when greater diagnostic specificity is clinically desired.

Previous studies have shown that gait speed is significantly slower in older adults with T2D than in those without diabetes [5]. In the present study, both UGS and MGS were lower in the sarcopenia group, suggesting that gait-related impairment may be more pronounced in participants with coexisting sarcopenia, even among older adults with T2D. This interpretation is supported by previous findings showing further gait-related impairment in individuals with coexisting sarcopenia [33]. However, because evidence directly comparing these relationships in non-diabetic populations remains limited, the extent to which the present findings can be generalized beyond older adults with T2D remains unclear and warrants further investigation.

Furthermore, previous studies have suggested that MGS and GSR may be associated with mortality in several clinical populations. In patients with cardiovascular disease, maximal gait speed has been identified as an independent prognostic predictor, and its combined evaluation with usual gait speed has been shown to improve prognostic risk stratification [22]. In addition, gait reserve has been associated with mortality among patients undergoing hemodialysis [24]. These findings suggest that reduced gait reserve capacity represents impaired physiological resilience affecting long-term outcomes across disease populations, supporting the clinical relevance of MGS and GSR.

An important strength of this study is the systematic comparison of three complementary gait-related indices within the same population. These measurements require minimal equipment, can be performed quickly, and are easily implemented in routine clinical practice. Notably, the combined use of UGS and GSR supports the practical value of simple gait assessment in routine clinical settings.

Although gait speed has been removed from the diagnostic criteria in the updated AWGS 2025 definition [9], gait performance remains an important indicator of physical function and physiological reserve in older adults. Therefore, examining gait-related indices such as UGS, MGS, and GSR may provide complementary information for identifying individuals who are more likely to have sarcopenia beyond the current diagnostic framework.

Nevertheless, this study has several limitations. First, it focused on older adults with T2D; therefore, the applicability of the findings to populations without diabetes remains unclear. Second, this was a single-center study, which may limit external validity. Third, because of the cross-sectional design, causal relationships between gait indices and sarcopenia cannot be established. Fourth, the cutoff values for UGS, MGS, and GSR were derived from the same cohort in which diagnostic performance was evaluated, and therefore may not be directly generalizable to other populations. Fifth, although the diagnosis of sarcopenia in this study was based on AWGS 2025, future revisions to the diagnostic criteria for sarcopenia may affect the results of this study. Sixth, measurement errors associated with gait assessment and BIA may have influenced the results. This is because reliability coefficients and minimal detectable change were not evaluated in the present study. Future multicenter longitudinal studies are needed to confirm these findings and to determine whether gait reserve is associated with long-term clinical outcomes. Finally, SARC-F was not assessed in this study; therefore, direct comparison with established screening tools was not possible. Future studies should evaluate gait-based indices alongside widely used questionnaires such as SARC-F to clarify their relative utility.

## 5. Conclusions

In older adults with T2D, UGS, MGS, and GSR were independently associated with sarcopenia. UGS showed high sensitivity, whereas GSR showed high specificity, suggesting that these indices may play complementary roles in the identification of sarcopenia. In addition, the combination of UGS and GSR improved specificity-related diagnostic performance compared with UGS alone.

These findings suggest that gait-related indices may provide complementary information for identifying older adults with T2D who are more likely to have sarcopenia. However, given the moderate diagnostic performance and the cross-sectional design, further studies are needed to confirm their clinical utility.

## Figures and Tables

**Table 1:** Clinical and physical characteristics of participants with type 2 diabetes with or without sarcopenia.

	Total (*n* = 117)	Sarcopenia (*n* = 32)	Non-Sarcopenia (*n* = 85)	*p*-Value
Age, years	73.0 (69.0–77.0)	76.0 (73.0–82.0)	72.0 (69.0–75.0)	<0.001
Male, *n* (%)	68 (58.1)	18 (56.3)	50 (58.8)	0.836
BMI, kg/m^2^	23.2 (21.5–26.2)	21.8 (20.2–22.7)	24.6 (22.0–27.3)	<0.001
Duration of diabetes, years	10.0 (2.0–20.0)	12.0 (2.8–20.3)	8.5 (1.0–18.0)	0.296
HbA1c, %	9.2 (8.0–10.8)	9.3 (8.0–10.4)	9.2 (8.0–10.8)	0.843
eGFR (mL/min/1.73 m^2^)	66.9 (49.4–79.5)	66.3 (48.1–78.9)	67.0 (51.0–79.5)	0.702
Diabetic neuropathy, *n* (%)	50 (42.7)	19 (59.4)	31 (36.5)	0.036
Diabetic retinopathy, *n* (%)	35 (29.9)	14 (43.8)	21 (24.7)	0.069
Diabetic nephropathy, *n* (%)	40 (34.2)	12 (37.5)	28 (32.9)	0.667
Hypertension, *n* (%)	80 (68.4)	21 (65.6)	59 (69.4)	0.824
Dyslipidemia, *n* (%)	51 (43.6)	12 (37.5)	39 (45.9)	0.531
Cerebrovascular disease, *n* (%)	9 (7.7)	3 (9.4)	6 (7.1)	0.704
Cardiovascular disease, *n* (%)	18 (15.4)	3 (9.4)	15 (17.6)	0.391
SMI, kg/m^2^	6.71 (6.06–7.23)	5.83 (5.42–6.60)	7.03 (6.28–7.54)	<0.001
HGS, kg	23.8 (18.5–30.3)	18.3 (14.2–23.8)	26.5 (21.0–31.8)	<0.001
UGS, m/s	1.03 (0.90–1.17)	0.93 (0.80–1.03)	1.10 (0.96–1.21)	<0.001
MGS, m/s	1.35 (1.14–1.56)	1.13 (0.90–1.27)	1.45 (1.29–1.63)	<0.001
GSR, m/s	0.33 (0.17–0.43)	0.17 (0.08–0.29)	0.35 (0.27–0.45)	<0.001

Values are presented as median (interquartile range) or number (%). *p* values indicate comparisons between the Sarcopenia and Non-sarcopenia groups. BMI, body mass index; SMI, skeletal muscle mass index; HGS, handgrip strength; UGS, usual gait speed; MGS, maximal gait speed; GSR, gait speed reserve.

**Table 2:** Multivariable logistic regression analysis for factors associated with sarcopenia.

	Model 1			Model 2			Model 3		
	OR	95% CI	*p*-value	OR	95% CI	*p*-value	OR	95% CI	*p*-value
Age (years)	1.08	0.98–1.20	0.119	1.07	0.96–1.19	0.198	1.10	0.99–1.21	0.074
Sex (male = 1, female = 0)	1.64	0.54–5.01	0.386	2.77	0.80–9.65	0.109	2.03	0.65–6.36	0.225
BMI (kg/m^2^)	0.74	0.63–0.87	<0.001	0.71	0.60–0.84	<0.001	0.73	0.62–0.86	<0.001
Diabetic neuropathy (yes)	1.37	0.45–4.16	0.576	1.38	0.44–4.35	0.579	1.30	0.44–3.85	0.639
UGS (per 0.1 m/s)	0.54	0.38–0.75	<0.001						
MGS (per 0.1 m/s)				0.58	0.45–0.75	<0.001			
GSR (per 0.1 m/s)							0.43	0.28–0.66	<0.001

Outcome variable: sarcopenia (1 = sarcopenia, 0 = non-sarcopenia). Model 1 included UGS; Model 2 included MGS; Model 3 included GSR. All models were adjusted for age, sex, BMI, and diabetic neuropathy. HGS and SMI were excluded because they are components of the diagnostic criteria for sarcopenia. Gait speed variables were entered into the models per 0.1 m/s increase. OR, odds ratio; CI, confidence interval; BMI, body mass index; UGS, usual gait speed; MGS, maximal gait speed; GSR, gait speed reserve.

**Table 3:** Diagnostic performance of gait-related indices for identifying sarcopenia.

	Cutoff Values (m/s)	Sensitivity (%)	Specificity (%)	Accuracy (%)	PPV (%)	NPV (%)	PLR	NLR
UGS	1.07	90.6	55.3	65.0	43.3	94.0	2.0	0.2
MGS	1.28	75.0	78.8	77.8	57.1	89.3	3.5	0.3
GSR	0.21	65.6	83.5	78.6	60.0	86.6	4.0	0.4
UGS + GSR	<1.07 & <0.21	68.8	89.4	83.8	71.0	88.4	6.5	0.4

Cutoff values for UGS and MGS were determined using ROC analysis with the Youden index. GSR was calculated as MGS minus UGS. The combined criterion was defined as UGS < 1.07 m/s and GSR < 0.21 m/s. UGS, usual gait speed; MGS, maximal gait speed; GSR, gait speed reserve; PPV, positive predictive value; NPV, negative predictive value; PLR, positive likelihood ratio; NLR, negative likelihood ratio.

## Data Availability

The data are not publicly available due to ethical restrictions but may be available from the corresponding author upon reasonable request and with permission from the Ethics Committee.

## Abbreviations

The following abbreviations are used in this manuscript:

AWGS	Asian Working Group for Sarcopenia
GSR	Gait speed reserve
HGS	Handgrip strength
MGS	Maximal gait speed
SMI	Skeletal muscle mass index
T2D	Type 2 diabetes
UGS	Usual gait speed
